# Identification and analysis of key genes associated with acute myocardial infarction by integrated bioinformatics methods

**DOI:** 10.1097/MD.0000000000025553

**Published:** 2021-04-16

**Authors:** Siyu Guo, Jiarui Wu, Wei Zhou, Xinkui Liu, Yingying Liu, Jingyuan Zhang, Shanshan Jia, Jialin Li, Haojia Wang

**Affiliations:** Department of Clinical Chinese Pharmacy, School of Chinese Materia Medica, Beijing University of Chinese Medicine, Beijing 100102, China.

**Keywords:** acute myocardial infarction, bioinformatics, biomarkers, differentially expressed gene

## Abstract

**Background:**

Acute myocardial infarction (AMI) is a common disease leading threat to human health around the world. Here we aimed to explore new biomarkers and potential therapeutic targets in AMI through adopting integrated bioinformatics tools.

**Methods:**

The gene expression Omnibus (GEO) database was used to obtain genes data of AMI and no-AMI whole blood. Furthermore, differentially expressed genes (DEGs) were screened using the “Limma” package in R 3.6.1 software. Functional and pathway enrichment analyses of DEGs were performed via “Bioconductor” and “GOplot” package in R 3.6.1 software. In order to screen hub DEGs, the STRING version 11.0 database, Cytoscape and molecular complex detection (MCODE) were applied. Correlation among the hub DEGs was evaluated using Pearson's correlation analysis.

**Results:**

By performing DEGs analysis, 289 upregulated and 62 downregulated DEGs were successfully identified from GSE66360, respectively. And they were mainly enriched in the terms of neutrophil activation, immune response, cytokine, nuclear factor kappa-B (NF-κB) signaling pathway, IL-17 signaling pathway, and tumor necrosis factor (TNF) signaling pathway. Based on the data of protein–protein interaction (PPI), the top 10 hub genes were ranked, including interleukin-8 (CXCL8), TNF, N-formyl peptide receptor 2 (FPR2), growth-regulated alpha protein (CXCL1), transcription factor AP-1 (JUN), interleukin-1 beta (IL1B), platelet basic protein (PPBP), matrix metalloproteinase-9 (MMP9), toll-like receptor 2 (TLR2), and high affinity immunoglobulin epsilon receptor subunit gamma (FCER1G). What's more, the results of correlation analysis demonstrated that there was positive correlation between the 10 hub DEGs.

**Conclusion:**

Ten DEGs were identified as potential candidate diagnostic biomarkers for patients with AMI in present study. However, further experiments are needed to confirm the functional pathways and hub genes associated with AMI.

## 1 Introduction

According to the report of World Health Organization (WHO), cardiovascular diseases (CVDs) are modern epidemic, and it accounts for most noncommunicable diseases death.^[1]^ With significant advances in adjunctive pharmacotherapy and revascularization strategies of acute myocardial infarction (AMI), the mortality of AMI has improved.^[2,3]^ However, AMI is still the leading threat to human health in CVDs.^[4,5]^ In addition, previous studies have shown that the short-term prognosis of AMI is poor.^[6,7]^ The cause of AMI is mainly related to the rupture of atherosclerotic plaque, leading to myocardial hypoxia, necrosis, and extensive myocardial damage.^[8]^ The risk factors associated with AMI are mainly caused by the interaction between genetic and environmental factors, including diabetes mellitus, hypercholesterolemia, obesity, and hypertension.^[9]^ Therefore, an in-depth understanding of AMI is great significance.

At present, the key treatment strategies for AMI include reducing myocardial oxygen demand, pharmacotherapy, thrombolysis, and percutaneous coronary intervention.^[10]^ However, these treatment methods may cause some adverse effects. For example, combination of various antithrombotic drugs will increase the incidence of bleeding complications.^[11]^ Furthermore, compared with thrombolytic therapy, primary PCI seems to be more effective for acute ST-segment elevation myocardial infarction.^[12]^ Overall, in order for AMI patients to be treated as soon as possible, early diagnosis is essential.

In recent years, several serum biomarkers were widely used to identify AMI patients, such as creatine kinase myocardial band (CK-MB), cardiac troponin (cTn) isoforms I and T.^[13]^ Although CK-MB and cTn are 2 major biomarkers for early diagnosis of AMI, the false positive rates and the risks associated with detecting a single biomarker have also become troubling problems.^[14,15]^ In order to better assist in the diagnosis of AMI, it is also necessary to explore new biomarkers in the blood.

With the advancement of high-throughput gene chip and transcriptome sequencing technology, integrated bioinformatics provides an effective tool for discovering valuable new biomarker targets. Using bioinformatics strategy, Shen et al found that fibronectin (FN1), neuromedin-U (NMU), chordin-like protein (CHRDL1), guanine nucleotide-binding protein subunit alpha-14 (GNA14), vasopressin V1a receptor (AVPR1A), guanine nucleotide-binding protein G(i) subunit alpha-1 (GNAI1), and integrin alpha-2 (ITGA2) may be potential biomarkers for the treatment of thyroid cancer.^[16]^ Zou et al clarified that zinc finger protein 566 (ZNF566), zinc finger homeobox protein 3 (ZFHX3), PDZK1-interacting protein 1 (PDZK1IP1), and pituitary homeobox 2 (PITX2) are significantly associated with new biomarkers of atrial fibrillation-related stroke through the bioinformatics analysis of gene expression Omnibus (GEO) dataset.^[17]^ Besides, Qian et al analyzed the microarray data of GSE59867 and found that proto-oncogene c-Fos (FOS), integrin alpha-IIb (ITGA2B), thrombospondin-1 (THBS1), and interleukin-8 (CXCL8) may play a vital role in development of heart failure after acute ST-segment elevation myocardial infarction.^[18]^ However, there is still a large amount of bioinformatics data related to AMI to be explored.

**Figure 1:**
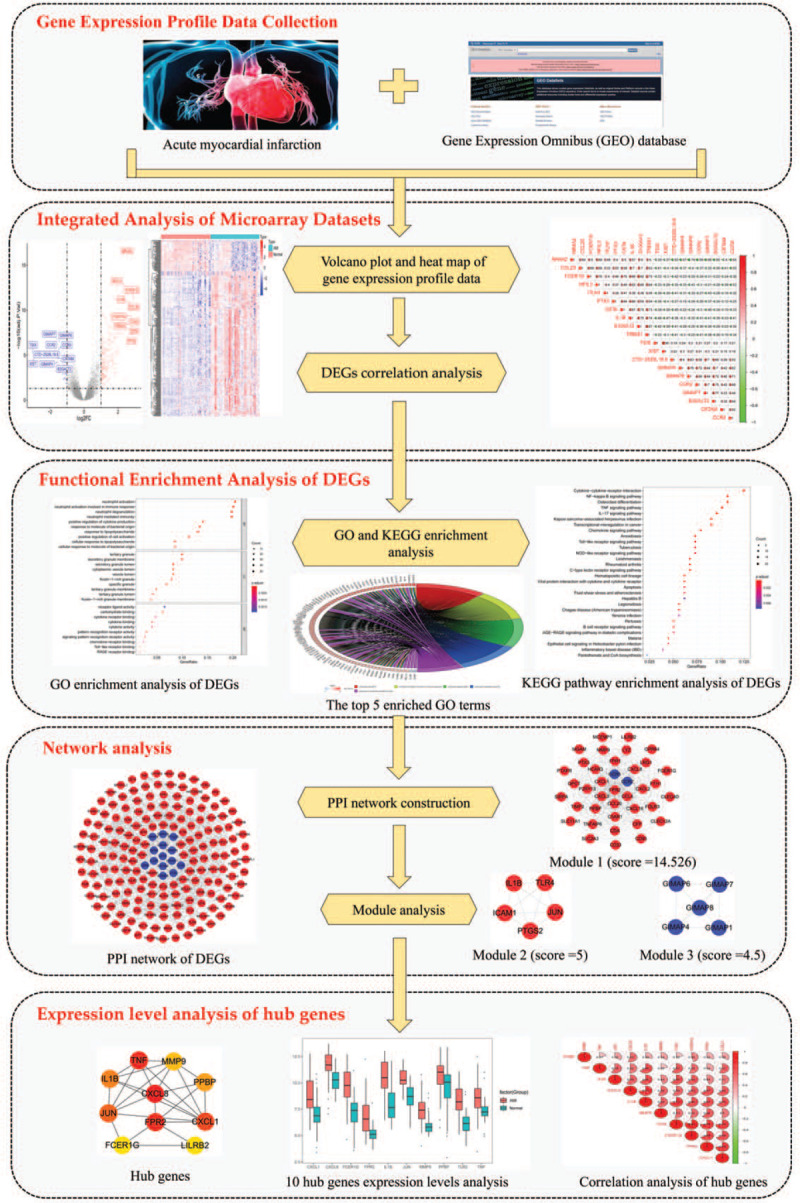
The detailed workflow for identification of key potential genes associated with acute myocardial infarction.

In this study, the microarray data of GSE66360 were applied to identify the differentially expressed genes (DEGs) between AMI and non-AMI whole blood utilizing an integrated bioinformatics. Afterwards, in order to analyze the main biological functions regulated by DEGs, enrichment analysis was conducted. Meanwhile, through correlation analysis, protein–protein interaction (PPI) and modular analysis of upregulated DEGs, key genes related to the diagnosis and treatment of AMI were identified. The detailed workflow is shown in Figure 1.

## 2 Materials and methods

### 2.1 Gene expression profile data

The gene expression profile data of GSE66360, downloaded from GEO database (https://www.ncbi.nlm.nih.gov/gds/),^[19]^ were used to screen DEGs in AMI. Ninety-nine specimens (49 AMI samples and 50 control samples) were included in the data series of GSE66360. The blood collection time was shortly after the AMI patients arrived at the acute care facility, and their arterial blood was collected. In addition, the age of the research population was 18 to 80 years old, both genders. And the gene expression was detected by GPL570 [HG-U133_Plus_2] Affymetrix Human Genome U133 Plus 2.0 Array.

### 2.2 Screening of DEGs and correlation analysis

Using the “Limma” package in R 3.6.1 software,^[20]^ normalization, log 2 conversion and DEGs screening of GSE66360 matrix data were performed. |log2FC| > 1 and adjust *P*-value < .05 were regarded as the statistical significance threshold level of DEGs samples.^[21,22]^ In addition, to get a better understanding of DEGs, correlation analysis was applied using Pearson's correlation and the visualized by adopting “corrplot” R package.^[23,24]^

### 2.3 Functional enrichment analysis of DEGs

The statistically significant DEGs were further analyzed in R 3.6.1 software with the “Bioconductor” and “GOplot” package,^[25,26]^ to conduct the Gene Ontology (GO) function and Kyoto Encyclopedia of Genes and Genomes (KEGG) pathway enrichment analyses.^[27,28]^ Adjust *P*-value < .05 was regarded as the cut-off criteria.

### 2.4 PPI network construction and module analysis

In order to obtain directly or indirectly interacting proteins related to DEGs, the STRING version 11.0 database (https://string-db.org/) was used.^[29]^ At the same time, the confidence score was set as >0.7 and species limited to “Homo sapiens.” Subsequently, the Cytoscape version 3.7.1 (http://www.cytoscape.org/) plug-in the molecular complex detection (MCODE) was used to identify the key modules from PPI network.^[21]^ And modules with MCODE score >4.5 were presented.

### 2.5 Expression level analysis of hub genes

By using the boxplot tool in ImageGP (http://www.ehbio.com/ImageGP/) to show the difference in expression of hub genes in AMI and no-AMI whole blood.

## 3 Results

### 3.1 Identification of DEGs

**Figure 2:**
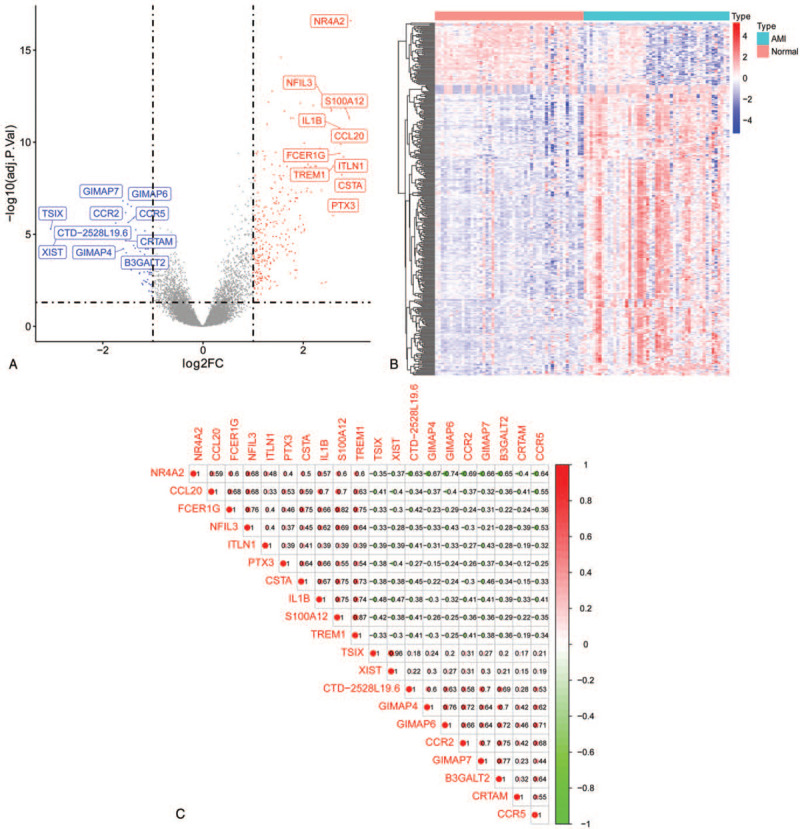
(A) Volcano plot of GSE66360. Blue ones represented downregulation, red ones indicated upregulation, and gray ones were the rest of the DEGs. (B) Heat map of DEGs. Each column represents one dataset and each row indicates one gene. Blue represents downregulated genes and red represents upregulated genes. (C) Bitmap of the correlation analysis between the top 10 up- or downregulated DEGs. Red and green denoted positive and negative correlation, respectively. DEGs: differentially expressed genes.

GSE66360 was selected and underwent DEGs analysis using “Limma” package in R 3.6.1 software. Three hundred fifty-one DEGs were identified either up- or downregulated in all, including 289 up- and 62 downregulated genes (|log2FC| > 1 and adjust *P*-value < .05) (Supplementary file 1). As shown in Figure 2A, all 351 DEGs were plotted that blue ones represented downregulation, red ones indicated upregulation, and gray ones were the rest of the DEGs. What's more, the expression levels of all the DEGs were presented in the heatmap (Fig. 2B), and these genes were well clustered between AMI and control group.

### 3.2 Correlation analysis

The top 10 up- or downregulated DEGs were selected for correlation analysis (|log2FC| > 1 and adjust *P*-value < .05). Figure 2C shows a bitmap of the correlation analysis between the DEGs. Red and green denoted positive and negative correlation, respectively. And the darker the color, the higher the correlation coefficient. According to the classification for Pearson's correlation coefficient (*r*), the absolute value of 0 to 0.30, 0.30 to 0.50, 0.50 to 0.70, and 0.70 to 1.00 represented “poor” correlation, “fair” or “moderate” correlation, “good” correlation, and “strong” correlation, respectively. Furthermore, “*r* = 0” indicated “no correlation at all” and “*r* = 1.00” represented “perfect correlation.”^[30]^ As listed in Figure 2C, XIST had a notable positive correlation with TSIX (*r* = 0.96). However, XIST had highly negative correlation with NFIL3 (*r* = −0.28). In addition, a stronger negative correlation existed between PTX3 and CRTAM (*r* = −0.12). TREM1 and S100A12 also had relatively obvious positive correlation with interleukin-1 beta (IL1B) (*r* = 0.74 and *r* = 0.75).

### 3.3 Functional enrichment analysis of DEGs

**Figure 3:**
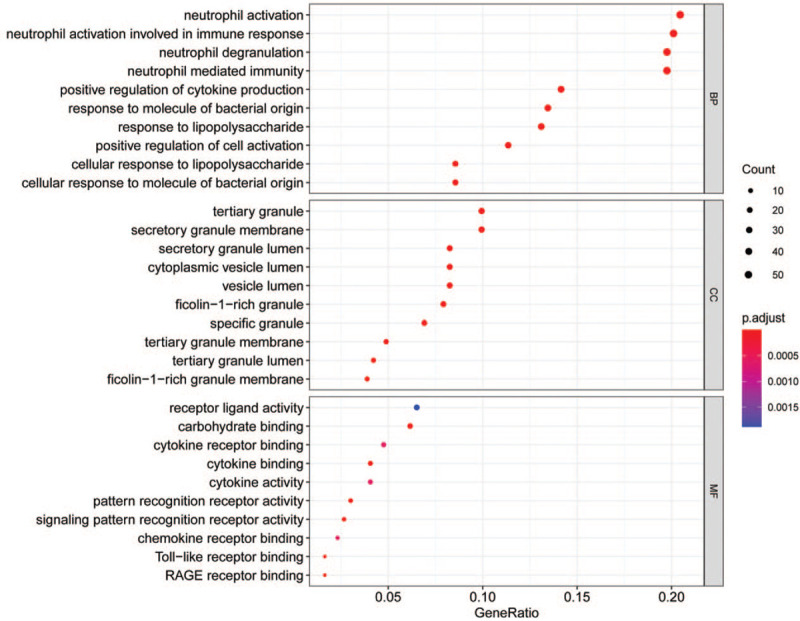
GO enrichment analysis of DEGs (*P*-value < .01 and *q*-value < 0.05). DEGs: differentially expressed genes; GO: Gene Ontology.

**Figure 4:**
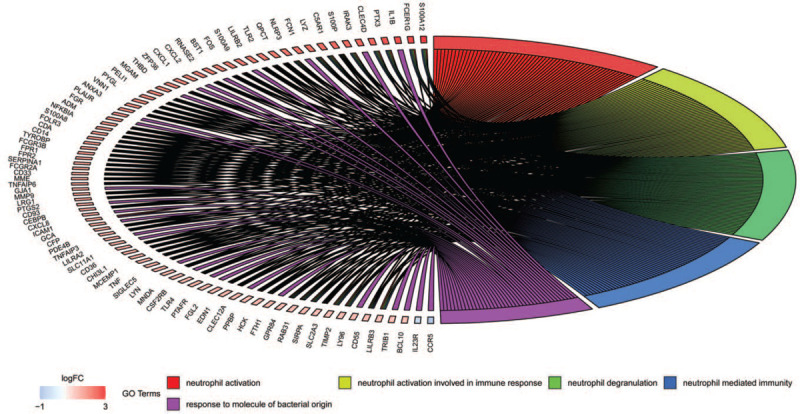
The top 5 enriched GO terms. GO: Gene Ontology.

**Figure 5:**
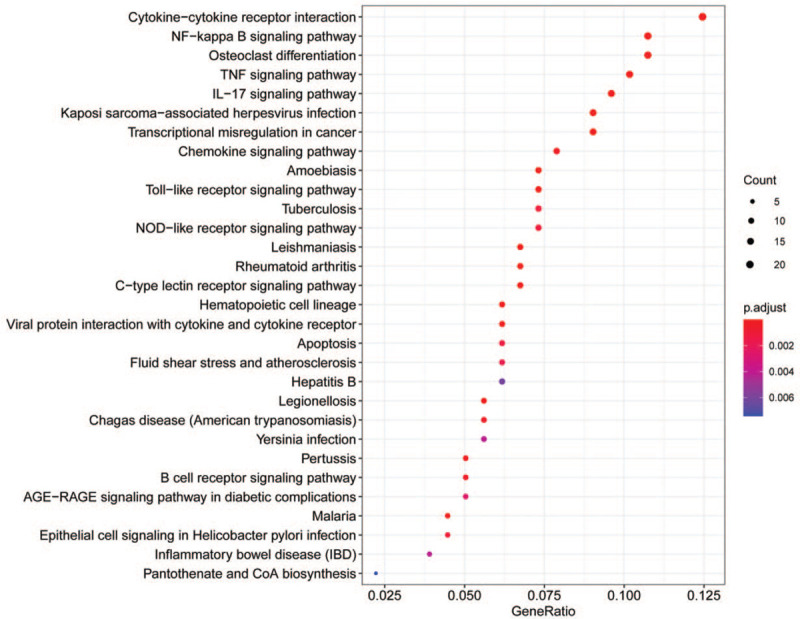
KEGG pathway enrichment analysis of DEGs (*P*-value < .05 and *q*-value < 0.05). DEGs: differentially expressed genes; KEGG: Kyoto Encyclopedia of Genes and Genomes.

To obtain a deeper insight into the biological functions of DEGs, GO annotation and KEGG pathway enrichment analyses were performed. The top 10 enriched GO terms were shown in Figures 3 and 4. The GO terms were comprised of 3 parts: cellular component (CC), biological process (BP), and molecular function (MF).^[31]^ DEGs of BP were involved in neutrophil activation, neutrophil mediated immunity, response to molecule of bacterial origin, lipopolysaccharide, positive regulation of cytokine production, and cell activation. CC analysis revealed that DEGs were markedly enriched tertiary granule, secretory granule membrane, tertiary granule lumen, and cytoplasmic vesicle lumen. For MF analysis, the top 3 significantly enriched terms were receptor ligand activity, carbohydrate binding, and cytokine receptor binding. Besides, the enriched KEGG pathways as presented in Figure 5, including cytokine–cytokine receptor interaction, Nuclear factor kappa-B (NF-κB) signaling pathway, osteoclast differentiation, tumor necrosis factor (TNF) signaling pathway, and IL-17 signaling pathway.

### 3.4 PPI network construction and module analysis

**Figure 6:**
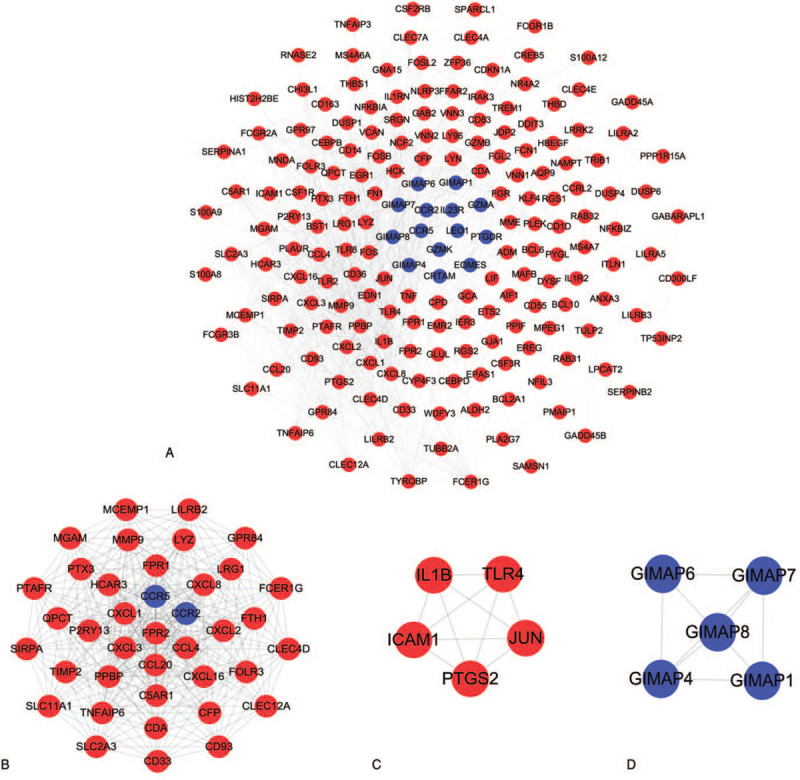
Analysis of DEGs PPI network and module analysis. (A) PPI network of DEGs. (B) Module 1 contained 39 gene nodes and 279 edges, MCODE score = 14.526. (C) Module 2 contained 5 upregulated genes nodes and 10 edges, MCODE score = 5. (D) Module 3 contained 5 downregulated genes nodes and 9 edges, MCODE score = 4.5. Blue represents downregulated genes and red represents up-regulated genes. DEGs: differentially expressed genes; PPI: protein–protein interaction; MCODE: molecular complex detection.

The STRING database was used to identify the PPI pairs. As revealed in Figure 6A, 187 nodes (DEGs) and 778 edges (interactions) were established in the constructed PPI network. According to the degree value, the top 10 hub DEGs were determined. The results shown that CXCL8 was the most crucial gene with the highest degree = 38, followed by TNF at degree = 35, N-formyl peptide receptor 2 (FPR2) at degree = 35, growth-regulated alpha protein (CXCL1) at degree = 33, transcription factor AP-1 (JUN) at degree = 31, IL1B at degree = 30, platelet basic protein (PPBP) at degree = 29, matrix metalloproteinase-9 (MMP9) at degree = 26, toll-like receptor 2 (TLR2) at degree = 25, and high affinity immunoglobulin epsilon receptor subunit gamma (FCER1G) at degree = 25 (Table 1).

**Table 1:** The information of top 10 hub genes based on their degree value.

Gene name	Protein name	Expression level	Degree	Enriched significant modules
CXCL8	Interleukin-8	Up	38	Module 1
TNF	Tumor necrosis factor	Up	35	None
FPR2	N-formyl peptide receptor 2	Up	35	Module 1
CXCL1	Growth-regulated alpha protein	Up	33	Module 1
JUN	Transcription factor AP-1	Up	31	Module 2
IL1B	Interleukin-1 beta	Up	30	Module 2
PPBP	Platelet basic protein	Up	29	Module 1
MMP9	Matrix metalloproteinase-9	Up	26	Module 1
TLR2	Toll-like receptor 2	Up	25	None
FCER1G	High affinity immunoglobulin epsilon receptor subunit gamma	Up	25	Module 1

Furthermore, the 3 significant modules (score > 4.5) were extracted from the PPI network. Module 1 contained 39 gene nodes, including FPR2, CXCL1, PPBP, CXCL8, and CC motif chemokine 20 (CCL20) with 279 edges (Fig. 6B). Module 2 contained 5 upregulated genes nodes and 10 edges, including IL1B, intercellular adhesion molecule 1 (ICAM1), JUN, prostaglandin G/H synthase 2 (PTGS2), and toll-like receptor 4 (TLR4) (Fig. 6C). Module 3 contained 5 downregulated genes nodes and 9 edges, including GTPase IMAP family member 4 (GIMAP4), GIMAP7, GIMAP1, GIMAP6, and GIMAP8 (Fig. 6D). Notably, 6 hub DEGs namely CXCL8, FPR2, CXCL1, PPBP, MMP9, and FCER1G were found in module 1. Two hub DEGs of JUN and IL1B were enriched in module 2. However, TNF and TLR2 were not shown in significant modules.

### 3.5 Expression level analysis of hub genes

**Figure 7:**
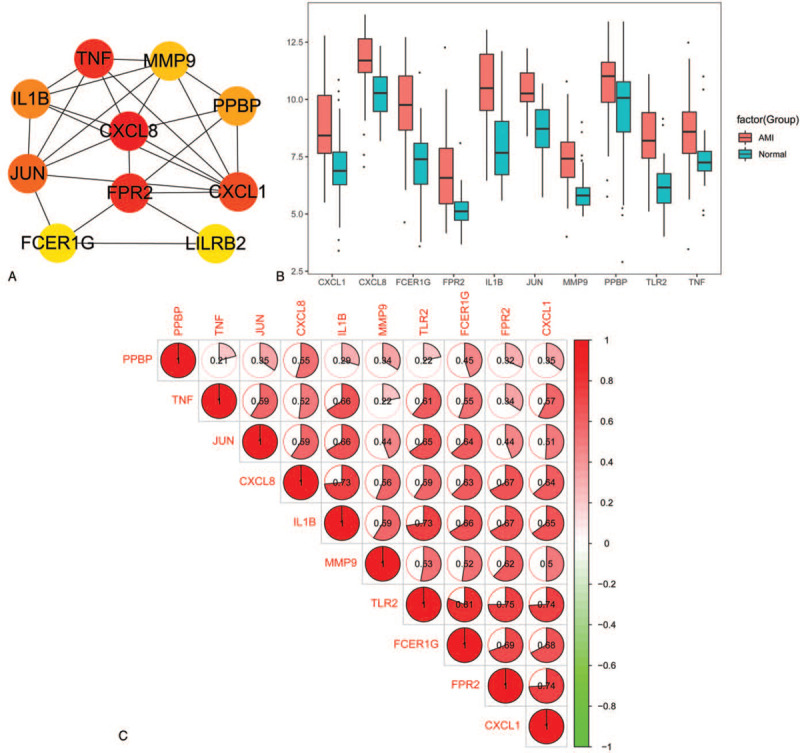
(A) The network of 10 hub genes. The 10 nodes are displayed from red (high degree value) to yellow (low degree value). (B) Analysis of 10 hub genes expression level in acute myocardial infarction. (C) Bitmap of the correlation analysis between the 10 hub genes. Red and green denoted positive and negative correlation, respectively.

The interaction network between the 10 hub DEGs was constructed by Cytoscape version 3.7.1 plug-in “cytoHubba” based on their degree (Fig. 7A). As demonstrated in Figure 7B, the hub genes expression levels of CXCL8, TNF, FPR2, CXCL1, JUN, IL1B, PPBP, MMP9, TLR2, and FCER1G were markedly upregulated in AMI whole blood compared to those in non-AMI whole blood. Furthermore, correlation analysis between the expression levels of the 10 hub genes (CXCL8, TNF, FPR2, CXCL1, JUN, IL1B, PPBP, MMP9, TLR2, and FCER1G) was performed by adopting Pearson's correlation analysis. The results demonstrated that there was positive correlation between the 10 hub genes expression (Fig. 7C). Obviously, TLR2 had a notable positive correlation with FCER1G (*r* = 0.81).

## 4 Discussion

Despite advances in adjunctive pharmacotherapy and revascularization strategies of AMI, it is still the leading threat to human health in CVDs.^[32]^ Thus, successful screening techniques and accurate diagnosis remain the great challenges for decreasing the incidence of AMI. In the present study, integrated bioinformatics analysis was used to identify the potential key genes related to AMI. By performing DEGs analysis, 289 upregulated and 62 downregulated DEGs were successfully identified (|log2FC| > 1 and adjust *P*-value < .05), respectively. To obtain a deeper insight into the biological functions of DEGs, GO annotation and KEGG pathway enrichment analyses were performed. Based on the data of PPI, the top 10 hub genes were ranked, including CXCL8, TNF, FPR2, CXCL1, JUN, IL1B, PPBP, MMP9, TLR2, and FCER1G. Notably, 6 hub DEGs namely CXCL8, FPR2, CXCL1, PPBP, MMP9, and FCER1G were found in module 1. Two hub DEGs of JUN and IL1B were enriched in module 2. However, TNF and TLR2 were not shown in significant modules.

Accordingly, the expression level and correlation of 10 hub genes were analyzed. The 10 hub genes expression levels were markedly upregulated in AMI whole blood compared to those in non-AMI whole blood. Obviously, TLR2 had a notable positive correlation with FCER1G (*r* = 0.81).

GO analysis showed that the DEGs were enriched in neutrophil activation, immune response, cytokine, secretory granule, and receptor ligand activity. KEGG pathway annotation analysis revealed that DEGs were mainly involved in NF-κB signaling pathway, IL-17 signaling pathway, and TNF signaling pathway. However, the NF-κB signaling pathway was the most significantly enriched pathway. NF-κB was regarded as a crucial transcription factor in plaque destabilization.^[33]^ Previous studies have indicated the NF-κB family (p50, p52, p65, c-Rel, and Rel B) plays a crucial role in inflammatory process by promoting the expression of pro-inflammatory factors.^[34]^ In this study, 4 hub DEGs, namely IL1B, CXCL1, CXCL8, and TNF, enriched in NF-κB signaling pathway.

IL-1B, which is mainly produced by the activation of innate immune cells, is one of the classical interleukin-1 (IL-1) cytokines.^[35,36]^ And IL-1B has been considered as an important pro-inflammatory cytokine.^[37,38]^ Previous animal studies have demonstrated that IL-1 is linked with the development mechanisms of atherosclerosis.^[39]^ In arterial lesions, IL-1 stimulates vascular smooth muscle cells through transforming adherence molecules and growth factor-β, leading to coagulation and thrombosis.^[40]^ What's more, levels of IL-1B are increased in patients with AMI.^[41]^ Furthermore, the IL1B-3737T polymorphism modifies a binding site of the anti-inflammatory p50 subunit, which belongs to the NF-κB transcription factor family.^[42]^ IL-1B also induces the synthesis of inflammatory mediators associated with atherosclerotic plaque formation, such as C-reactive protein and interleukin-6.^[40]^ Activation of the NLRP3-inflammasome pathway and production of IL-1B after cellular damage caused by infarct is a key process in AMI. Veltman predicted IL-1B activation kinetics based on Ordinary Differential Equation model, and the study found that by attenuating the production of IL-1B proline, more upstream regulation was more potent in attenuating active IL-1B production than direct inhibition of the NLRP3-inflammasome.^[43]^

Recent evidence indicates that CXC chemokine is not only the key to inflammation, but also mediates the recruitment of inflammatory leukocytes.^[44–46]^ Pordel et al conducted that the plasma levels of CXCL1 chemokine were notably increased in AMI patients compared with healthy participants. And the plasma levels of CXCL1 were significantly correlated with TT genotype of TRAF3IP2 (rs33980500) in myocardial infarction patients.^[47]^ Whereas, CXCL8 may mediate the recruitment and activation of neutrophils, and promote the formation of new blood vessels.^[48]^ In addition, CXCL8 is upregulated in the infarct area inducing polymorphonuclear leukocyte infiltration.^[49]^ It was reported that CXCL1 and CXCL8 participate in pathogenic processes of several cancers, such as colorectal cancer, non-small cell lung cancer, and gastric carcinoma.^[50–52]^

In the experimental model of animal myocardial infarction, expression of TNF is markedly and consistently increased.^[53]^ TNF can promote inflammation damage, inducing the synthesis of chemokines, and adhesion molecules in the infarcted myocardium.^[54]^ It is worth noting that TNF signaling gives rise to cytoprotective signals that delay or prevent the development of cardiac myocyte apoptosis.^[55]^ Previous research suggested some plasma-induced cardiac biomarkers, such as TNF, IL6, MMP9, and cell-adhesion molecules could be valuable in the diagnosis and prognosis of AMI.^[56]^ The NF-κB transcriptional activation pathway is regarded as the “master regulator” of inflammation.^[57]^ Activated NF-κB increases the expression of TNF-α, IL-6, and IL-1β, thereby activating collagen deposition and myocardial fibrosis, leading to heart failure and myocardial remodeling.^[58,59]^ Similarly, FPR2, JUN, PPBP, MMP9, TLR2, and FCER1G expression has significant diagnosis value in AMI patients and acts as potential targets for AMI-targeted therapy.^[60–63]^ Consistent with these results, the present study indicated that IL1B, CXCL1, CXCL8, TNF, FPR2, JUN, PPBP, MMP9, TLR2, and FCER1G were upregulated hub DEGs.

Some limitations exist in this study. First, the sample size of the microarray data set used in this study is relatively small. Second, although we have identified some enriched pathways and core DEGs, the hierarchical processes between them have not yet been fully elucidated. However, in order to identify potential biomarkers associated with AMI, further studies with larger sample sizes are still warranted.

## 5 Conclusion

In conclusion, the present study identified IL1B, CXCL1, CXCL8, TNF, FPR2, JUN, PPBP, MMP9, TLR2, and FCER1 as key genes in the pathogenesis of AMI by integrated analysis of microarray datasets. The results of this study further provide useful evidence for investigation into molecular mechanisms, selection of biomarkers, and treatment targets exploration of AMI. However, further vitro and in vivo analyses experiments are needed to confirm the functional pathways and hub genes associated with AMI.

## Author contributions

GSY and WJR conceived and designed the study. ZW, LXK, and JSS collected the data. LYY, ZJY, LJL, and WHJ performed the data analysis, and GSY wrote the manuscript. All authors were responsible for reviewing data. All authors read and approved the final manuscript.

**Conceptualization:** Siyu Guo, Jiarui Wu.

**Data curation:** Jiarui Wu, Wei Zhou, Jialin Li, Haojia Wang.

**Formal analysis:** Wei Zhou.

**Methodology:** Xinkui Liu.

**Validation:** Jingyuan Zhang, Shanshan Jia.

**Visualization:** Jingyuan Zhang, Shanshan Jia.

**Writing – original draft:** Siyu Guo.

**Writing – review & editing:** Yingying Liu.

## Supplementary Material

Supplemental Digital Content
