# Supplementary material for: Identification and analysis of key genes associated with acute myocardial infarction by integrated bioinformatics methods
Source: Medicine (Baltimore). 2021 Apr 16;100(15):e25553. doi: 10.1097/MD.0000000000025553 (PMC8052032; doi:10.1097/MD.0000000000025553)
Supplement: Supplemental Digital Content [file medi-100-e25553-s001.docx]

Supplementary file 1. 289 up-regulated genes and 62 down-regulated genes in AMI compared with non-AMI whole blood.

| Gene | Log2FC | Adjust *P* Value | Expression level |
| --- | --- | --- | --- |
| NR4A2 | 2.952248 | 2.52E-17 | Up |
| GABARAPL1 | 1.558021 | 2.47E-15 | Up |
| NFKBIZ | 1.386029 | 1.72E-13 | Up |
| PDE4B | 1.312245 | 6.91E-13 | Up |
| THBD | 1.947923 | 7.36E-13 | Up |
| IRAK3 | 2.371404 | 8.44E-13 | Up |
| ACSL1 | 2.205522 | 1.01E-12 | Up |
| IL1R2 | 2.541951 | 1.01E-12 | Up |
| NFIL3 | 2.573047 | 1.95E-12 | Up |
| MAP3K8 | 1.632794 | 2.06E-12 | Up |
| ZFP36 | 1.955159 | 3.12E-12 | Up |
| PELI1 | 1.929161 | 3.12E-12 | Up |
| CLEC4E | 1.806793 | 4.88E-12 | Up |
| BCL6 | 1.757406 | 4.88E-12 | Up |
| S100A12 | 2.928184 | 5.16E-12 | Up |
| NAMPT | 2.024149 | 9.36E-12 | Up |
| SLC11A1 | 1.288006 | 1.05E-11 | Up |
| IL1B | 2.729049 | 1.69E-11 | Up |
| PPP1R15A | 2.030444 | 1.12E-10 | Up |
| CCL20 | 2.752548 | 1.26E-10 | Up |
| S100P | 2.342775 | 2.12E-10 | Up |
| ITPRIP | 1.205776 | 3.05E-10 | Up |
| HBEGF | 1.094899 | 3.29E-10 | Up |
| FCN1 | 2.185369 | 3.29E-10 | Up |
| JUN | 1.707199 | 3.43E-10 | Up |
| NLRP3 | 2.152413 | 3.43E-10 | Up |
| VCAN | 2.475511 | 3.43E-10 | Up |
| GADD45A | 1.455879 | 3.63E-10 | Up |
| CLEC4D | 2.414193 | 3.69E-10 | Up |
| FCER1G | 2.730116 | 4.01E-10 | Up |
| FOSL2 | 1.023352 | 4.01E-10 | Up |
| ADIPOR1 | 1.081284 | 4.30E-10 | Up |
| MAFB | 2.01929 | 5.29E-10 | Up |
| NR4A3 | 1.651104 | 5.41E-10 | Up |
| GLUL | 1.248051 | 5.65E-10 | Up |
| LILRB2 | 2.102924 | 5.65E-10 | Up |
| ITLN1 | 2.813325 | 6.20E-10 | Up |
| JDP2 | 1.236763 | 8.18E-10 | Up |
| NFKBIA | 1.78449 | 1.09E-09 | Up |
| PLAUR | 1.846045 | 1.16E-09 | Up |
| PMAIP1 | 1.626555 | 1.19E-09 | Up |
| TLR2 | 2.103763 | 1.63E-09 | Up |
| IL1RN | 1.381797 | 1.63E-09 | Up |
| SERPINA1 | 1.544797 | 1.93E-09 | Up |
| TREM1 | 2.607962 | 1.93E-09 | Up |
| LYZ | 2.268683 | 1.96E-09 | Up |
| TP53INP2 | 2.23982 | 1.96E-09 | Up |
| CDA | 1.685795 | 1.98E-09 | Up |
| QPCT | 2.147864 | 2.22E-09 | Up |
| SLC7A7 | 2.045394 | 2.35E-09 | Up |
| HAL | 1.226343 | 2.93E-09 | Up |
| IER3 | 2.061753 | 3.15E-09 | Up |
| S100A9 | 2.102492 | 3.15E-09 | Up |
| CCL4 | 2.120214 | 4.99E-09 | Up |
| CXCL2 | 1.973948 | 5.10E-09 | Up |
| C5AR1 | 2.340306 | 5.50E-09 | Up |
| CD83 | 2.250492 | 5.50E-09 | Up |
| CSTA | 2.779091 | 5.85E-09 | Up |
| GAB2 | 1.417311 | 5.85E-09 | Up |
| CLEC7A | 1.536011 | 6.57E-09 | Up |
| SNORD89 | 1.613697 | 6.57E-09 | Up |
| CD55 | 1.046422 | 6.57E-09 | Up |
| THBS1 | 1.553829 | 8.50E-09 | Up |
| SULF2 | 1.567752 | 1.05E-08 | Up |
| PHACTR1 | 1.888425 | 1.05E-08 | Up |
| BST1 | 2.070264 | 1.16E-08 | Up |
| TRIB1 | 1.039948 | 1.16E-08 | Up |
| ETS2 | 1.44145 | 1.51E-08 | Up |
| AIF1 | 1.596778 | 3.01E-08 | Up |
| GADD45B | 1.36582 | 3.10E-08 | Up |
| ALDH2 | 2.091124 | 3.16E-08 | Up |
| ICAM1 | 1.388083 | 3.22E-08 | Up |
| TULP2 | 1.772645 | 3.87E-08 | Up |
| AQP9 | 2.374881 | 4.07E-08 | Up |
| FOS | 2.076311 | 4.60E-08 | Up |
| FCGR2A | 1.539046 | 4.86E-08 | Up |
| CSF3R | 2.164826 | 5.07E-08 | Up |
| CMTM2 | 1.969052 | 5.12E-08 | Up |
| MAFF | 1.555367 | 5.12E-08 | Up |
| CPD | 1.105537 | 5.40E-08 | Up |
| DYSF | 1.860675 | 5.45E-08 | Up |
| FOSB | 1.707238 | 6.71E-08 | Up |
| IL13RA1 | 1.481611 | 1.03E-07 | Up |
| PYGL | 1.893749 | 1.06E-07 | Up |
| VNN1 | 1.856943 | 1.08E-07 | Up |
| CXCL16 | 1.878121 | 1.12E-07 | Up |
| SKIL | 1.404684 | 1.12E-07 | Up |
| TP53BP2 | 1.134661 | 1.45E-07 | Up |
| PDZD8 | 1.072607 | 1.56E-07 | Up |
| EFEMP1 | 1.481132 | 1.56E-07 | Up |
| DDIT3 | 1.754135 | 1.58E-07 | Up |
| CCRL2 | 1.740882 | 1.58E-07 | Up |
| BCL2A1 | 1.846533 | 1.68E-07 | Up |
| CH25H | 1.429821 | 1.95E-07 | Up |
| LRG1 | 1.439898 | 1.97E-07 | Up |
| MGP | 1.516245 | 2.01E-07 | Up |
| MMP9 | 1.463947 | 2.21E-07 | Up |
| SIRPA | 1.078501 | 2.66E-07 | Up |
| CDKN1A | 1.630674 | 2.81E-07 | Up |
| LILRA5 | 1.118283 | 3.06E-07 | Up |
| RGS1 | 1.269211 | 3.18E-07 | Up |
| DUSP1 | 1.015952 | 3.18E-07 | Up |
| PPIF | 1.231474 | 3.51E-07 | Up |
| KLF4 | 1.37147 | 4.01E-07 | Up |
| FPR1 | 1.610761 | 4.18E-07 | Up |
| RBP7 | 1.728 | 4.31E-07 | Up |
| PILRA | 1.57356 | 4.98E-07 | Up |
| MME | 1.500871 | 5.60E-07 | Up |
| ZEB2 | 1.195037 | 5.71E-07 | Up |
| DOCK4 | 1.115273 | 6.78E-07 | Up |
| FAM198B | 1.406472 | 7.50E-07 | Up |
| CD33 | 1.533814 | 8.22E-07 | Up |
| WDFY3 | 1.230208 | 8.85E-07 | Up |
| DOCK5 | 1.043382 | 9.11E-07 | Up |
| PTX3 | 2.604807 | 9.22E-07 | Up |
| MGAM | 1.943165 | 9.55E-07 | Up |
| TNFAIP2 | 1.225491 | 1.06E-06 | Up |
| AC079305.10 | 1.947447 | 1.38E-06 | Up |
| METRNL | 1.129695 | 1.46E-06 | Up |
| SLC2A3 | 1.060796 | 1.47E-06 | Up |
| SYTL3 | 1.047916 | 1.59E-06 | Up |
| LILRA2 | 1.294171 | 1.63E-06 | Up |
| CSRNP1 | 1.154737 | 1.63E-06 | Up |
| GLT1D1 | 1.634969 | 1.66E-06 | Up |
| RLF | 1.103082 | 1.92E-06 | Up |
| MS4A6A | 1.462321 | 2.02E-06 | Up |
| LINC00528 | 1.412054 | 2.16E-06 | Up |
| RAB32 | 1.354967 | 2.28E-06 | Up |
| FPR2 | 1.60176 | 2.44E-06 | Up |
| TLR4 | 1.164965 | 2.62E-06 | Up |
| SLC7A5 | 1.272735 | 2.71E-06 | Up |
| SAMSN1 | 1.481873 | 2.71E-06 | Up |
| PLBD1 | 2.009553 | 2.91E-06 | Up |
| VNN3 | 1.111049 | 3.07E-06 | Up |
| CD36 | 1.282808 | 3.07E-06 | Up |
| SECTM1 | 1.267907 | 3.65E-06 | Up |
| CD93 | 1.423983 | 3.74E-06 | Up |
| P2RY13 | 1.923005 | 3.90E-06 | Up |
| MBOAT2 | 1.061768 | 4.87E-06 | Up |
| GNA15 | 1.052468 | 5.00E-06 | Up |
| PTAFR | 1.156505 | 5.21E-06 | Up |
| CPVL | 1.852222 | 5.23E-06 | Up |
| LPCAT2 | 1.22661 | 5.38E-06 | Up |
| HCAR3 | 2.033916 | 5.44E-06 | Up |
| MXD1 | 1.093774 | 5.55E-06 | Up |
| ANXA3 | 1.849155 | 5.69E-06 | Up |
| EREG | 1.702673 | 5.83E-06 | Up |
| C9orf72 | 1.121821 | 6.29E-06 | Up |
| RNASE2 | 2.025886 | 6.68E-06 | Up |
| GPR84 | 1.085028 | 7.32E-06 | Up |
| SAT1 | 1.14206 | 7.72E-06 | Up |
| CXCL1 | 1.970999 | 7.95E-06 | Up |
| TMCC3 | 1.18368 | 8.58E-06 | Up |
| CD163 | 1.576517 | 9.37E-06 | Up |
| CD14 | 1.66729 | 9.61E-06 | Up |
| MCEMP1 | 1.248753 | 1.10E-05 | Up |
| RNF175 | 1.469387 | 1.30E-05 | Up |
| CXCL8 | 1.405308 | 1.31E-05 | Up |
| EPAS1 | 1.005007 | 1.34E-05 | Up |
| CD300LF | 1.727413 | 1.40E-05 | Up |
| EMR2 | 1.11483 | 1.48E-05 | Up |
| IFNGR1 | 1.023661 | 1.57E-05 | Up |
| DMXL2 | 1.546975 | 1.58E-05 | Up |
| LY96 | 1.056667 | 1.73E-05 | Up |
| FCGR3B | 1.657392 | 1.95E-05 | Up |
| BCL10 | 1.021248 | 2.01E-05 | Up |
| GPR97 | 1.177815 | 2.21E-05 | Up |
| CHI3L1 | 1.264269 | 2.76E-05 | Up |
| ARHGEF40 | 1.076151 | 2.85E-05 | Up |
| ADM | 1.802668 | 3.01E-05 | Up |
| ARL5B | 1.065113 | 3.30E-05 | Up |
| VNN2 | 1.330839 | 3.54E-05 | Up |
| FOLR3 | 1.698946 | 3.59E-05 | Up |
| HAUS3 | 1.206475 | 3.62E-05 | Up |
| KCTD12 | 1.558914 | 3.77E-05 | Up |
| DDX3Y | 1.489959 | 3.85E-05 | Up |
| TM6SF1 | 1.596041 | 4.43E-05 | Up |
| SCML1 | 1.142771 | 4.51E-05 | Up |
| CYP4F3 | 1.375907 | 4.69E-05 | Up |
| RP6-99M1.2 | 1.599308 | 4.76E-05 | Up |
| CEBPD | 1.196888 | 5.57E-05 | Up |
| LYN | 1.198552 | 6.18E-05 | Up |
| RNF144B | 1.074077 | 6.27E-05 | Up |
| SLC8A1-AS1 | 1.150577 | 6.30E-05 | Up |
| MPP1 | 1.346366 | 6.30E-05 | Up |
| TYROBP | 1.664655 | 6.74E-05 | Up |
| RP11-443B7.1 | 1.026686 | 7.48E-05 | Up |
| RAB31 | 1.084496 | 7.56E-05 | Up |
| FURIN | 1.070266 | 8.03E-05 | Up |
| G0S2 | 1.27561 | 8.53E-05 | Up |
| TIMP2 | 1.058379 | 8.53E-05 | Up |
| S100A8 | 1.768269 | 8.77E-05 | Up |
| C15orf48 | 1.732275 | 8.87E-05 | Up |
| TNFAIP6 | 1.492641 | 0.000102 | Up |
| MPEG1 | 1.842483 | 0.000105 | Up |
| SULF1 | 1.338725 | 0.000107 | Up |
| DUSP6 | 1.192428 | 0.000122 | Up |
| KCNJ2 | 1.686827 | 0.000131 | Up |
| DUSP4 | 1.11262 | 0.000134 | Up |
| IDI2-AS1 | 1.012653 | 0.000142 | Up |
| OGN | 1.179557 | 0.000142 | Up |
| EDN1 | 1.139859 | 0.000143 | Up |
| RELT | 1.027985 | 0.000166 | Up |
| CLEC4A | 1.311796 | 0.000167 | Up |
| FN1 | 1.017025 | 0.000171 | Up |
| FAM49A | 1.133936 | 0.000174 | Up |
| UTY | 1.335433 | 0.000181 | Up |
| CLEC12A | 1.108923 | 0.000188 | Up |
| CTB-31O20.2 | 1.486634 | 0.000191 | Up |
| LILRB3 | 1.040458 | 0.000206 | Up |
| TNFAIP3 | 1.30561 | 0.000206 | Up |
| CEBPB | 1.415603 | 0.000225 | Up |
| HIST2H2BE | 1.231577 | 0.000252 | Up |
| BRE-AS1 | 1.064957 | 0.000256 | Up |
| TMEM176A | 1.187441 | 0.000259 | Up |
| RASGEF1B | 1.096109 | 0.000261 | Up |
| SIGLEC5 | 1.233129 | 0.00028 | Up |
| ALDH1A1 | 1.425605 | 0.000281 | Up |
| SLC15A3 | 1.113071 | 0.000281 | Up |
| FGR | 1.821195 | 0.000286 | Up |
| NRGN | 1.284907 | 0.00029 | Up |
| SLC31A2 | 1.178763 | 0.000311 | Up |
| LOC645984 | 1.351359 | 0.000345 | Up |
| KIAA1598 | 1.118874 | 0.000362 | Up |
| LOC731424 | 1.091888 | 0.000366 | Up |
| GCA | 1.354475 | 0.00037 | Up |
| AMPD2 | 1.063473 | 0.000384 | Up |
| CXCL3 | 1.362449 | 0.000397 | Up |
| RP11-373D23.2 | 1.276218 | 0.000403 | Up |
| LGALS2 | 1.121357 | 0.00047 | Up |
| TNF | 1.247905 | 0.000522 | Up |
| FCGR1B | 1.24944 | 0.00055 | Up |
| MARCKS | 1.026071 | 0.000691 | Up |
| FFAR2 | 1.121311 | 0.000769 | Up |
| ARRDC4 | 1.334191 | 0.000778 | Up |
| CFP | 1.341916 | 0.000798 | Up |
| EIF1AY | 1.878471 | 0.000864 | Up |
| TXLNGY | 1.849777 | 0.00089 | Up |
| RPH3A | 1.026414 | 0.000934 | Up |
| CSF1R | 1.08201 | 0.000988 | Up |
| SGK1 | 1.139731 | 0.00109 | Up |
| FTH1 | 1.092395 | 0.001156 | Up |
| ZNF331 | 1.057549 | 0.001189 | Up |
| PLEK | 1.005077 | 0.001208 | Up |
| HCK | 1.09724 | 0.001218 | Up |
| IRS2 | 1.097221 | 0.001256 | Up |
| GZMB | 1.746033 | 0.001293 | Up |
| PLA2G7 | 1.170632 | 0.001376 | Up |
| KLF10 | 1.151967 | 0.001408 | Up |
| ZNF185 | 1.004546 | 0.001836 | Up |
| EGR1 | 1.081271 | 0.001842 | Up |
| EGR3 | 1.302746 | 0.001896 | Up |
| TTTY15 | 1.32857 | 0.001931 | Up |
| RGS2 | 1.186531 | 0.001942 | Up |
| LIF | 1.013373 | 0.002085 | Up |
| SPARCL1 | 1.24788 | 0.002094 | Up |
| IER5 | 1.135836 | 0.002104 | Up |
| USP9Y | 1.851973 | 0.002305 | Up |
| CSF2RB | 1.190478 | 0.002329 | Up |
| TLR8 | 1.152821 | 0.002487 | Up |
| LOC101927069 | 1.259058 | 0.0025 | Up |
| CTC-510F12.4 | 1.111136 | 0.002592 | Up |
| CTA-29F11.1 | 1.174489 | 0.002613 | Up |
| DNMBP | 1.097345 | 0.00274 | Up |
| CD1D | 1.290942 | 0.002906 | Up |
| FGL2 | 1.151849 | 0.003175 | Up |
| RTN1 | 1.031021 | 0.003327 | Up |
| NCF2 | 1.181507 | 0.003386 | Up |
| mir-223 | 1.354883 | 0.003566 | Up |
| PTGS2 | 1.43472 | 0.003816 | Up |
| RPS4Y1 | 2.459173 | 0.003965 | Up |
| CREB5 | 1.060436 | 0.00401 | Up |
| OR52K3P | 1.103828 | 0.004016 | Up |
| KDM5D | 2.376861 | 0.004504 | Up |
| PFKFB3 | 1.122155 | 0.00451 | Up |
| ZFY | 1.079462 | 0.005145 | Up |
| CLEC1A | 1.022247 | 0.006028 | Up |
| GJA1 | 1.483345 | 0.006095 | Up |
| SRGN | 1.014346 | 0.007119 | Up |
| LRRK2 | 1.350332 | 0.007454 | Up |
| MOP-1 | 1.039398 | 0.008121 | Up |
| MNDA | 1.190532 | 0.008456 | Up |
| LOC100130357 | 1.108466 | 0.008612 | Up |
| MS4A7 | 1.034762 | 0.008988 | Up |
| TUBB2A | 1.331943 | 0.009316 | Up |
| SLC22A4 | 1.08228 | 0.016017 | Up |
| SERPINB2 | 1.162065 | 0.017576 | Up |
| PPBP | 1.103833 | 0.036103 | Up |
| ASH1L-AS1 | 1.027508 | 0.041512 | Up |
| GIMAP7 | -1.59002 | 1.53E-07 | Down |
| GIMAP6 | -1.49444 | 2.30E-07 | Down |
| ZNF137P | -1.4246 | 3.06E-07 | Down |
| PAQR8 | -1.13339 | 5.60E-07 | Down |
| CCR2 | -1.53467 | 6.63E-07 | Down |
| CTD-2541M15.1 | -1.37567 | 1.29E-06 | Down |
| CCR5 | -1.48617 | 2.25E-06 | Down |
| CTD-2528L19.6 | -1.73212 | 2.45E-06 | Down |
| TSIX | -3.0357 | 5.15E-06 | Down |
| CISH | -1.33432 | 5.68E-06 | Down |
| ZNF420 | -1.154 | 6.49E-06 | Down |
| XIST | -2.88877 | 1.07E-05 | Down |
| GZMA | -1.06221 | 1.34E-05 | Down |
| RP11-214K3.19 | -1.1127 | 1.74E-05 | Down |
| CRTAM | -1.53437 | 2.21E-05 | Down |
| GCSAM | -1.07022 | 2.76E-05 | Down |
| RP11-96D1.11 | -1.05907 | 3.45E-05 | Down |
| LOC153682 | -1.37486 | 4.05E-05 | Down |
| EOMES | -1.34437 | 5.62E-05 | Down |
| GIMAP4 | -1.58017 | 6.09E-05 | Down |
| WDR86-AS1 | -1.08806 | 6.27E-05 | Down |
| GIMAP8 | -1.13326 | 6.30E-05 | Down |
| ZFP3 | -1.24475 | 8.53E-05 | Down |
| B3GALT2 | -1.52325 | 0.0001 | Down |
| RAD54B | -1.15787 | 0.000124 | Down |
| PTGDR | -1.00938 | 0.000155 | Down |
| RP11-747H7.3 | -1.03896 | 0.000194 | Down |
| IL23R | -1.10181 | 0.00022 | Down |
| LINC00260 | -1.1097 | 0.000236 | Down |
| GIMAP1 | -1.04219 | 0.000238 | Down |
| ELOVL4 | -1.24169 | 0.000249 | Down |
| DENND2D | -1.09754 | 0.00029 | Down |
| RP11-140I16.3 | -1.30836 | 0.00037 | Down |
| ZNF786 | -1.0635 | 0.000379 | Down |
| HMG20A | -1.06319 | 0.000392 | Down |
| VSIG1 | -1.11115 | 0.000505 | Down |
| GVINP1 | -1.13451 | 0.000549 | Down |
| ZNF260 | -1.06429 | 0.000606 | Down |
| TCEB3-AS1 | -1.04827 | 0.000812 | Down |
| AB488780 | -1.42518 | 0.000856 | Down |
| CTD-3025N20.3 | -1.10009 | 0.001068 | Down |
| GIN1 | -1.1678 | 0.001121 | Down |
| LEO1 | -1.06111 | 0.001172 | Down |
| POP5 | -1.0344 | 0.001177 | Down |
| RP11-722E23.2 | -1.0184 | 0.001218 | Down |
| AMIGO2 | -1.25236 | 0.001218 | Down |
| PRSS35 | -1.25917 | 0.001377 | Down |
| TNFAIP8L2 | -1.0645 | 0.001482 | Down |
| LINC00959 | -1.02824 | 0.00152 | Down |
| NOG | -1.09657 | 0.002906 | Down |
| A2M-AS1 | -1.1696 | 0.003229 | Down |
| TMEM60 | -1.14085 | 0.003746 | Down |
| GZMK | -1.11871 | 0.003899 | Down |
| LOC283357 | -1.20825 | 0.004665 | Down |
| GEMIN5 | -1.06696 | 0.004864 | Down |
| BTLA | -1.00926 | 0.005108 | Down |
| ZNF792 | -1.19421 | 0.005455 | Down |
| MEST | -1.039 | 0.005515 | Down |
| PKI55 | -1.008 | 0.006767 | Down |
| ZNF189 | -1.07029 | 0.011967 | Down |
| LINC00094 | -1.02401 | 0.012771 | Down |
| ZNF204P | -1.00859 | 0.02189 | Down |
